# Evaluation of cytokine expressions in patients with recurrent aphthous stomatitis: A systematic review and meta-analysis

**DOI:** 10.1371/journal.pone.0305355

**Published:** 2024-06-11

**Authors:** Fangjun Teng, Qiuchen Jin

**Affiliations:** Department of Stomatology, The Central Hospital of Wuhan, Tongji Medical College, Huazhong University of Science and Technology, Wuhan, Hubei Province, China; Jazan University Faculty of Dentistry, SAUDI ARABIA

## Abstract

This systematic review and meta-analysis aimed to evaluate the expression levels of various T helper (Th) cell-secreted cytokines in recurrent aphthous stomatitis (RAS). Case-control studies comparing the serum or salivary levels of cytokines between RAS patients and healthy controls were searched in PubMed, EMBASE, Web of Science, and Google Scholar prior to September 30, 2023. Cytokines produced by Th1 (interleukin [IL]-1, IL-2, IL-8, IL-12, tumor necrosis factor alpha [TNF-α], interferon gamma [IFN-γ]), Th2 (IL-4, IL-5, IL-6, IL-10, IL-13), and Th17 (IL-17A) cells were investigated. The standard mean difference (SMD) with 95% confidence interval (CI) was calculated to detect the difference. A total of 20 studies comprising 1070 RAS patients and 536 healthy controls were included. RAS patients had significantly higher salivary levels of IL-2 (SMD = 4.15, 95%CI 0.83–7.48), IL-5 (SMD = 0.53, 95%CI 0.05–1.00), IL-6 (SMD = 0.48, 95%CI 0.12–0.84), IL-12 (SMD = 0.94, 95%CI 0.18–1.71), and TNF-α (SMD = 1.31, 95%CI 0.44–2.18) compared to healthy controls. Serum levels of IL-6 (SMD = 0.48, 95%CI 0.30–0.66), TNF-α (SMD = 0.70, 95%CI 0.22–1.17), and IFN-γ (SMD = 0.72, 95%CI 0.17–1.28) were significantly increased, while serum IL-10 levels (SMD = -2.25, 95%CI -3.99 to -0.52) were reduced in RAS patients. Patients diagnosed with major RAS had markedly elevated serum IL-8 levels (SMD = 0.39, 95%CI 0.07–0.71) and a trend toward higher serum IL-6 levels (SMD = 0.51, 95%CI -0.02 to 1.04) than those with minor RAS. In conclusion, Th1/Th2-related cytokines, especially IL-2, IL-6, and TNF-α, are involved in the pathogenesis of RAS development and progression and are potential therapeutic targets for RAS.

## Introduction

Recurrent aphthous stomatitis (RAS), also known as recurrent aphthous ulcer (RAU), is the most common oral mucosal disease. It is characterized by recurrent episodes of painful ulcerations frequently found on the buccal or labial mucosa and even on the tongue [1]. It is highly prevalent, affecting up to one quarter of young adults, with a lifetime prevalence as high as 36.5% [2, 3]. RAS causes considerable pain and impacts eating, swallowing, and speaking, ultimately affecting the quality of life of patients [4]. RAS is a multifactorial, chronic, inflammatory, and ulcerative disease. Previous studies have identified genetic, immunologic, nutritional, microbial, emotional, hematological, and traumatic factors that predispose individuals to RAS [5–7]. However, the definitive pathogenesis mechanism of RAS remains largely unknown, posing significant challenges for dental practitioners in its treatment and management.

There is increasing evidence of oral epithelial destruction caused by a T lymphocyte-mediated immune response, indicating a close correlation between T lymphocytes and the pathogenesis of RAS [8]. Initial stimulation by antigens on mucosal keratinocytes induces T-lymphocyte activation, leading to the cascading release of various cytokines and the migration of lymphocytes and neutrophils [9]. This triggers a cytotoxic response, causing oral ulcerations. These cytokines are major mediators of the immune response and are secreted from a variety of T helper cells, including type 1 (Th1), type 2 (Th2), and type 17 (Th17) cells. Generally, Th1 cells produce pro-inflammatory cytokines that mediate cytotoxic immune response, such as interleukin (IL)-1, IL-2, IL-8, IL-12, tumor necrosis factor alpha (TNF-α), and interferon gamma (IFN-γ). Th2 cells release several anti-inflammatory cytokines, such as IL-4, IL-5, IL-6, IL-10, and IL-13, which promote B cell function and participate in humoral immunity. IL-17A, mainly secreted by Th17 cells, exerts a strong pro-inflammatory effect and induces the infiltration and tissue damage of inflammatory cells [10]. These cytokines play pivotal roles in regulating cell differentiation, proliferation, migration, and interaction, and they determine immune activation and tolerance [11].

Abnormal Th1/2/17 cytokine profiles are observed in RAS patients. Various cytokine levels, such as IL-2, IL-6, TNF-α, and IFN-γ, are significantly elevated in RAS patients [12–15], while IL-10 is produced less in RAS patients compared to healthy controls [16, 17]. These observations suggest that T-lymphocyte-mediated immunologic dysregulation is involved in the pathogenesis of RAS. However, an increasing number of comparative studies have detected significant variations in cytokine levels in RAS patients, and the role of cytokines in RAS pathogenesis remains inconclusive. These inconsistent results may be caused by variations in sample size, sample type, RAS type, disease phase, and detection method among comparative studies. To better understand the role of cytokines in RAS development and progression, we conducted a systematic review and meta-analysis to analyze the differences in Th1/Th2/Th17-related cytokine levels in serum and saliva samples between RAS patients and healthy controls.

## Materials and methods

### Literature search strategy

This meta-analysis was performed following the Preferred Reporting Items for Systematic review and Meta-analysis (PRISMA) guidelines (S1 Checklist) [18]. A comprehensive search was conducted in PubMed, EMBASE, Web of Science, and Google Scholar up to September 30, 2023. The search terms used in each database are listed in S1 Table. We did not restrict the language and manually reviewed reference lists of eligible studies and relevant reviews to identify additional candidate studies that were not captured in the comprehensive literature search.

### Inclusion and exclusion criteria

The eligibility of studies retrieved from the literature search was judged according to the following criteria:

- Patients diagnosed with RAS were included in the case group.
- Healthy controls without any oral mucosal or systemic disease were recruited in the control group.
- Concentrations of various cytokines, including interleukins, TNF-α, and IFN-γ, were determined in serum or saliva samples. Only cytokines reported by two or more studies were included for quantitative synthesis.

Ulcers associated with systemic diseases such as Behcet’s disease or Crohn’s disease were excluded. Case reports, reviews, meta-analyses, fundamental experimental studies, and studies without healthy controls or sufficient data were discarded.

### Quality assessment

The quality of eligible studies was assessed using the Newcastle-Ottawa Scale (NOS), which evaluates the risk of bias in three domains and assigns up to 9 stars [19]. The selection domain included four items assessing the risk of bias in selecting study groups. In our study, the comparability domain assessed whether the study groups were matched for age and sex. The exposure domain comprised three items for ascertaining exposure, i.e., concentrations of cytokines. Zero or one star was awarded for each item in the selection and exposure domains, whereas a maximum of two stars was assigned to the comparability domain. The total stars ranged from 0 to 9, with ≥7 stars representing high quality, 4–6 stars indicating medium quality, and <4 stars suggesting low quality.

### Data extraction

The following information from included studies was extracted: first author, country, publication year, type of RAS, sample type, disease stage at sample collection, matching factors for study groups, detection method of cytokines, sample size, age, sex, and detected cytokines. The mean and standard deviation (SD) values of cytokine concentrations in case and control groups were directly extracted when available or converted from median with interquartile and/or range values using methods introduced by Wan X *et al*. [20] and Luo D *et al*. [21].

Two authors (FT, QJ) independently conducted the literature search, selection of eligible studies, quality assessment, and data extraction. In case of conflict, consensus was reached through full discussion between these two authors.

### Statistical analysis

The I^2^ statistic was used to assess between-study heterogeneity. An I^2^ value greater than 50% indicated significant heterogeneity, leading to the application of a random-effect model for quantitative synthesis. Otherwise, a fixed-effect model was used. The standard mean difference (SMD) with 95% confidence interval (CI) was calculated using Cohen’s d index to compare cytokine levels in case and control groups. SMD values of <0.2, between 0.2 and 0.8, and >0.8 suggested small, moderate, and large effect sizes, respectively [22]. Differences in each cytokine were analyzed separately for saliva and serum samples. Further subgroup analyses were conducted, stratified by RAS type (minor, major, herpetiform), disease stage (active, remission), and detection method (enzyme-linked immunosorbent assay [ELISA], cytometric bead array [CBA]). Additionally, direct comparisons of cytokine levels were made among patients with different RAS types and at different disease stages. Sensitivity analysis using the Leave-One-Out method was also performed. Potential publication bias was assessed by examining the symmetry of the funnel plot and conducting Egger’s test. A P-value <0.05 indicated statistical significance. STATA v16.0 software (StataCorp., TX, US) was used for our meta-analysis.

### Certainty of evidence

Two authors (FT, QJ) independently assessed the certainty of evidence using the Grading of Recommendations Assessment, Development and Evaluation (GRADE) approach [23].The overall certainty of evidence was graded as very low, low, moderate, or high. In case of conflict, consensus was reached through full discussion.

## Results

### Baseline features of included studies

**Fig 1:**
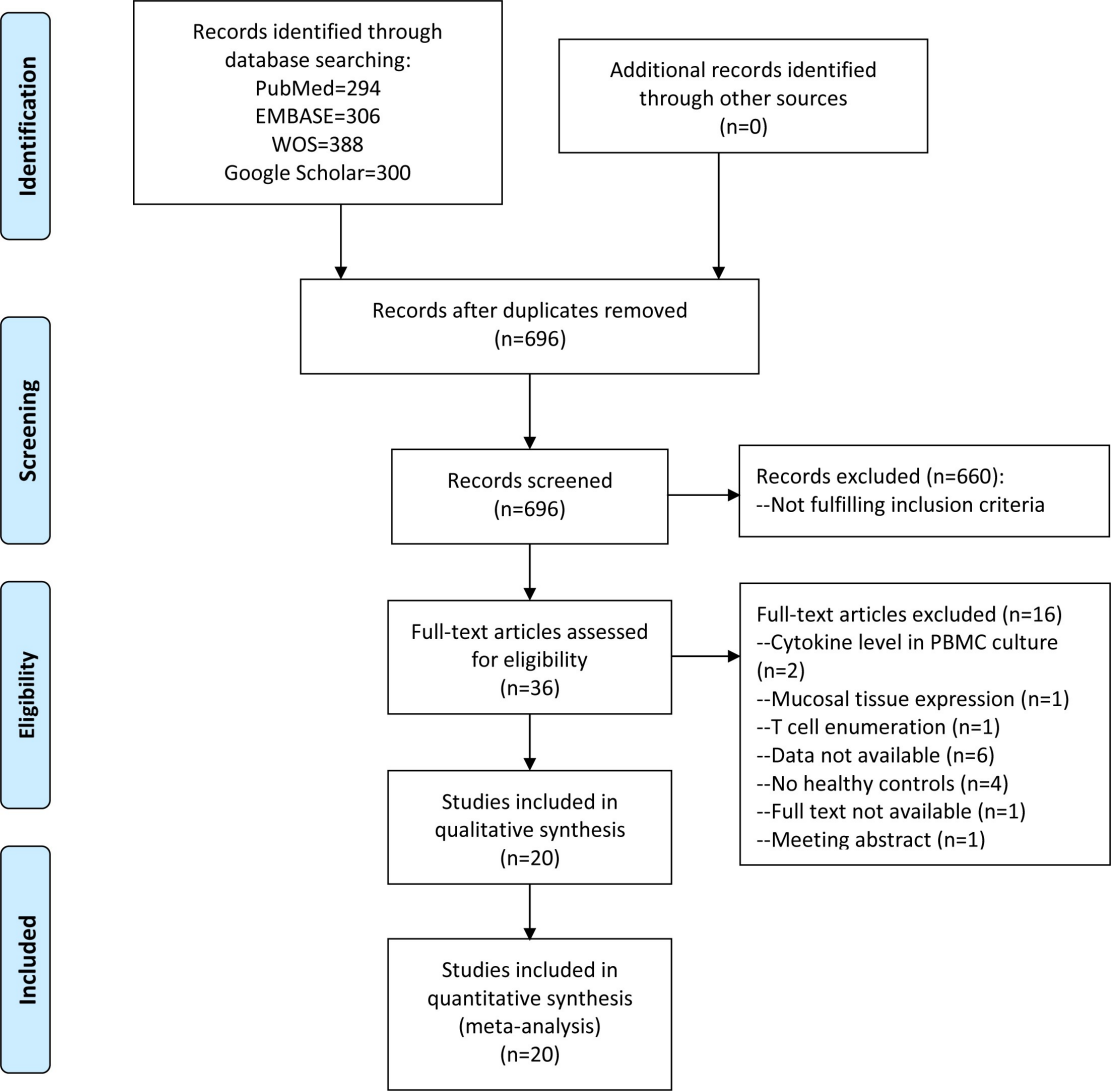
Flowchart of comprehensive literature search.

After removing 592 duplicates, a total of 696 unique articles underwent review for titles and abstracts, of which 660 were excluded based on inclusion/exclusion criteria. Among the remaining 36 records, 16 were discarded due to various reasons after full-text review (S2 Table). Finally, 20 studies comprising 1070 RAS patients and 536 healthy controls were included in our meta-analysis (Fig 1) [12–17, 24–37]. These studies were predominantly conducted in China (n = 5), India (n = 5), and Turkey (n = 5). The ages of RAS patients and controls exhibited wide ranges but were comparable between the two groups in most of the included studies. Sex distributions were also similar between the two groups in all studies except for 5 where sex distribution information was lacking. According to disease type, 6 studies exclusively recruited patients with minor RAS [16, 25, 26, 33, 35, 37], and 9 enrolled patients diagnosed with various types of RAS [13, 15, 17, 28–32, 34]. RAS types in the other 5 studies were not reported. One study collected both serum and saliva samples [26], 9 collected serum samples, and 10 collected saliva samples. Except for 2 studies collecting samples at both active and remission stages [25, 33], 7 studies detected cytokine levels at the active stage of RAS [14, 16, 27, 28, 31, 32, 35], and 2 at the remission stage [12, 26]. The disease phase at sample collection was not reported in the other studies. Cytokine concentrations were determined via ELISA method in 13 studies and CBA method in seven studies. IL-18 was excluded from quantitative analysis since it was detected in only one study [28]. Twelve cytokines, including IL-1 (n = 2), IL-2 (n = 7), IL-4 (n = 3), IL-5 (n = 2), IL-6 (n = 7), IL-8 (n = 3), IL-10 (n = 5), IL-12 (n = 4), IL-13 (n = 2), IL-17A (n = 4), TNF-α (n = 11), and IFN-γ (n = 6), were determined in two or more studies and then included in quantitative synthesis. The baseline features of studies included in the meta-analysis were summarized in Table 1.

**Table 1:** Baseline characteristics of studies included in meta-analysis.

Study	Country	RAS type	Disease phase	Sample	Method	RAS			Control			Cytokine	Reference
						No.	Age	Sex	No.	Age	Sex		
Sun A, 2003	China	Minor (113), major (68), herpetiform (16)	NA	Serum	ELISA	197	38 (5–72)	90/107	77	10–79	33/45	IL-6	15
Aridogan B, 2003	Turkey	NA	NA	Serum	ELISA	16	36.8±13.1	8/8	20	33.2±11.4	8/8	IL-4, IL-10, IL-12, IL-13, IFN-γ	24
Sun A, 2004	China	Minor (69), major (61), herpetiform (16)	Active	Serum	ELISA	146	39.4 (10–79)	66/80	54	10–69	24/30	IL-6, IL-8	31
Sun A, 2006	China	Minor (69), major (61), herpetiform (16)	Active	Serum	ELISA	146	39.4 (10–79)	66/80	54	10–69	24/30	TNF-α	32
Boras V, 2006	Croatia	Minor	Active, remission	Saliva	ELISA	26	27.3 (23–49)	NA	26	30.1 (22–64)	NA	IL-6, TNF-α	25
Borra R, 2009	Brazil	Minor	Remission	Serum, saliva	ELISA	17	31±8.5	7/10	17	33±10.6	7/10	TNF-α	26
Eguia-del Valle A, 2011	Spain	NA	Active	Saliva	ELISA	20	NA	NA	10	NA	NA	TNF-α	27
Pekiner F, 2012	Turkey	Minor, major	NA	Serum	CBA	30	37.03±13.84	12/18	15	30.26±8.98	9/6	IL-2, IL-6	30
Avci E, 2014	Turkey	Minor	Active	Serum	ELISA	25	28.9±3.8 ^a^	NA	25	NA	NA	IL-2, IL-10, IL-12, TNF-α	16
Ozyurt K, 2014	Turkey	Minor (21), major (3)	NA	Serum	ELISA	24	34.45±10.57	11/13	20	37±11.14	9/11	IL-1, IL-13, IL-17A, IFN-γ	29
Gupta P, 2014	India	Minor (18), major (10), herpetiform (2)	Active	Serum	ELISA	30	27.8±10.3	19/11	20	27.3±9.1	12/8	IL-8	28
Kalpana R, 2014	India	NA	Active	Saliva	ELISA	30	16–60	13/17	30	NA	12/18	IL-2	14
Seifi S, 2015	Iran	Minor (all)	Active, remission	Saliva	ELISA	18	31.5±10.7	5/13	18	NA	NA	IL-17A, TNF-α	33
Chaudhuri K, 2018	India	Minor (29), major (1)	NA	Saliva	ELISA	30	25.77±6.13	11/19	30	25.77±6.13	11/19	TNF-α	34
Bhosale S, 2018	India	Minor (16), major (1), herpetiform (3)	NA	Saliva	ELISA	20	20–60	10/10	20	20–60	10/10	IL-2, IL-10, IL-12	17
Hegde S, 2018	India	Minor (26), major (4)	NA	Saliva	ELISA	30	29.3±10.7	14/16	30	32.53±11.08	12/18	TNF-α	13
Shen C, 2021	China	Minor	NA	Serum	CBA	127	43.72±12.92	72/55	20	44.15±13.33	10/10	IL-2, IL-6, IL-17A, TNF-α, IFN-γ	37
Altay D, 2021	Turkey	NA	Remission	Saliva	ELISA	30	27.5 (20–44)	NA	25	23 (19–39)	NA	IL-2, IFN-γ	12
Novak T, 2021	UK	NA	NA	Saliva	ELISA	7	36.7±15	5/2	10	34.7±11.1	5/5	IL-1, IL-2, IL-4, IL-5, IL-6, IL-8, IL-10, IL-12, IL-17A, TNF-α, IFN-γ	36
Deng Y, 2022	China	Minor	Active	Saliva	CBA	101	49.7±12.2	53/48	15	47.1±7.5	7/8	IL-4, IL-5, IL-6, IL-10, TNF-α, IFN-γ	35

Age was indicated as mean±standard deviation or median and range.

Sex was indicated as number of males and females.

CBA: cytometric bead array; ELISA: enzyme-linked immunosorbent assay; NA: not available; RAS: recurrent aphthous stomatitis.

^a^ Mean age of all participants.

### Quality assessment of studies

According to NOS, only 9 studies were awarded one star for selection of controls, Age and sex, two factors potentially influencing cytokine levels, were not matched in 4 and 3 studies, respectively. All studies were awarded one star for each of the other 6 items. In total, all studies were assigned 7 or more stars and deemed as high-quality studies (S3 Table).

### IL-2 levels

**Fig 2:**
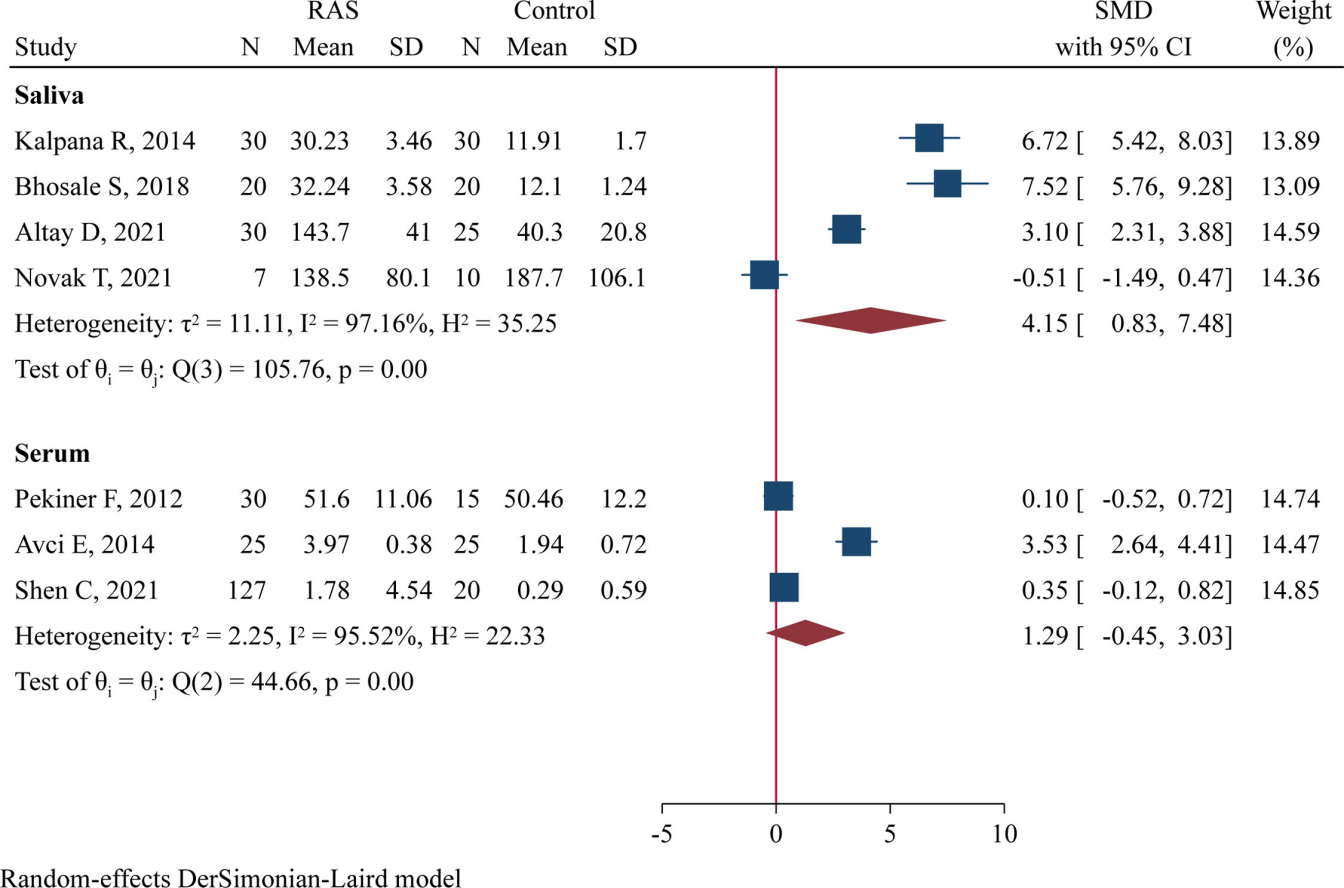
Forest plot of meta-analysis comparing IL-2 levels in saliva and serum samples between cases and controls.

The concentrations of salivary IL-2 were determined in 4 studies comprising 87 RAS and 85 controls [12, 14, 17, 36], and serum IL-2 levels were assessed in 3 studies involving 182 RAS and 60 controls [16, 30, 37]‥ Meta-analysis using a random-effect model revealed significantly higher salivary IL-2 levels in RAS patients compared to heathy controls (SMD = 4.15, 95%CI 0.83–7.48, P = 0.015, I^2^ = 97.2%; Fig 2). However, serum levels of IL-2 did not significantly differ between RAS patients and controls (SMD = 1.29, 95%CI -0.45 to 3.03, P = 0.147, I^2^ = 95.5%; Fig 2).

### IL-6 levels

**Fig 3:**
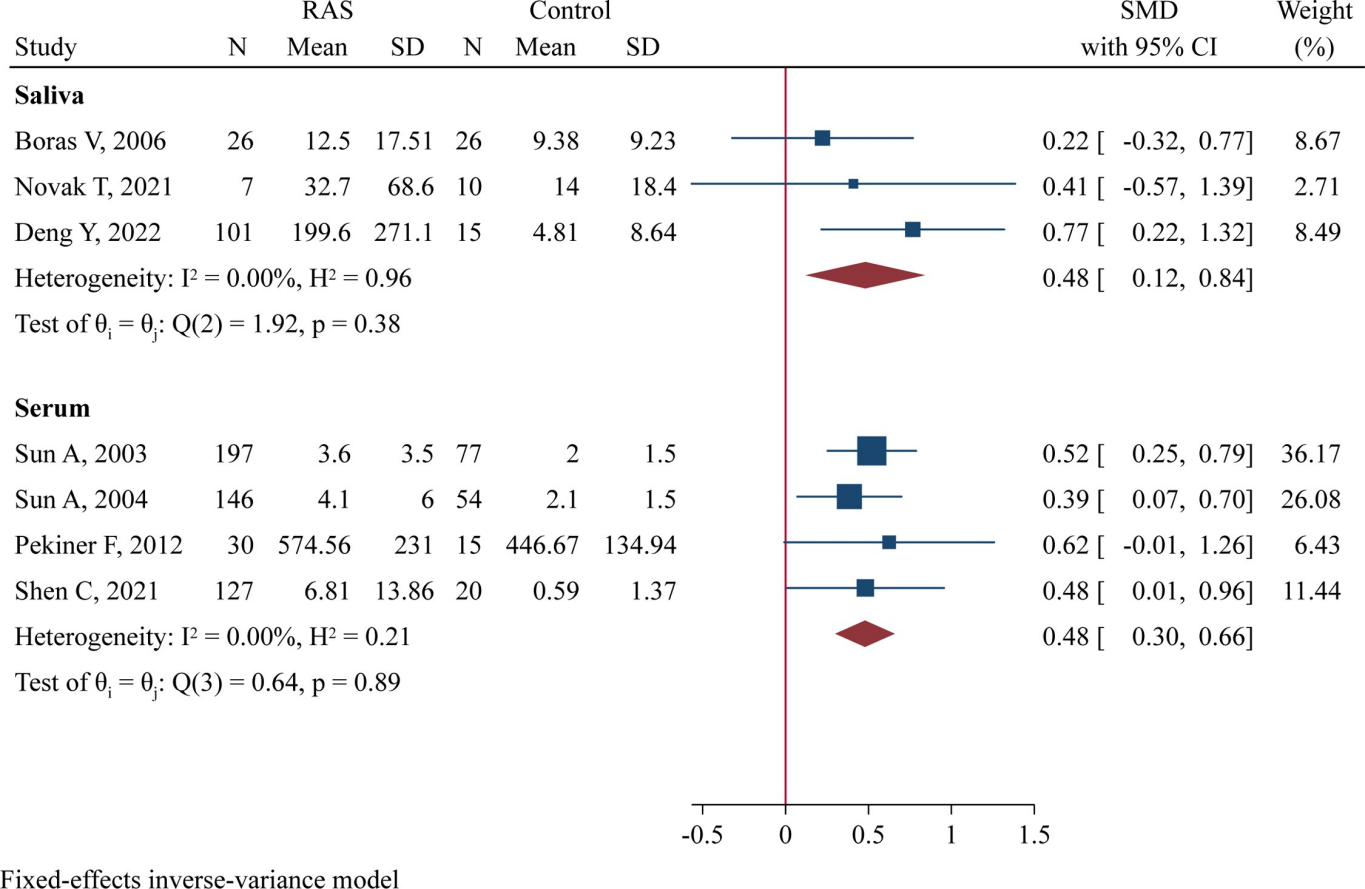
Forest plot of meta-analysis comparing IL-6 levels in saliva and serum samples between cases and controls.

There were 3 studies (134 RAS and 51 controls) assessing salivary IL-6 levels [25, 35, 36] and 4 studies (500 RAS and 166 controls) assessing serum IL-6 levels [15, 30, 31, 37]‥ There was no between-study heterogeneity, and a fixed-effect model was applied. Meta-analysis demonstrated that RAS patients had significantly higher IL-6 concentrations than healthy controls in both saliva samples (SMD = 0.48, 95%CI 0.12–0.84, P = 0.009; Fig 3) and serum samples (SMD = 0.48, 95%CI 0.30–0.66, P<0.001; Fig 3). The effect sizes were both moderate.

### IL-10 levels

**Fig 4:**
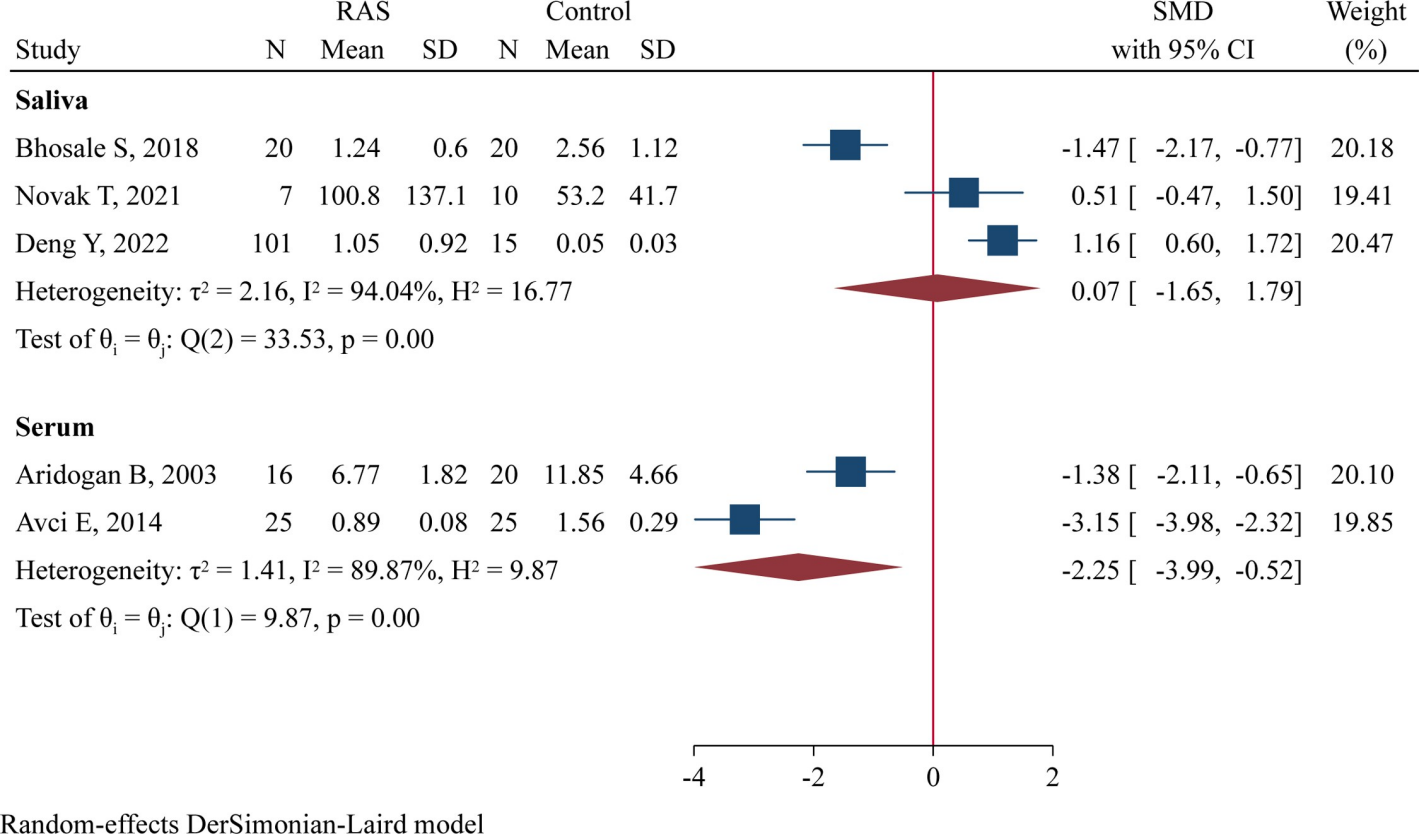
Forest plot of meta-analysis comparing IL-10 levels in saliva and serum samples between cases and controls.

Three studies including 128 RAS and 45 controls determined salivary IL-10 levels [17, 35, 36], and two studies comprising 41 RAS and 45 controls evaluated serum IL-10 concentrations [16, 24]. Significantly lower serum IL-10 concentrations were observed in RAS patients compared to healthy controls (SMD = -2.55, 95%CI -3.99 to -0.52, P = 0.011; Fig 4), but no difference in salivary IL-10 levels was noted between the two groups (SMD = 0.07, 95%CI -1.65 to 1.79, P = 0.938; Fig 4).

### TNF-α levels

**Fig 5:**
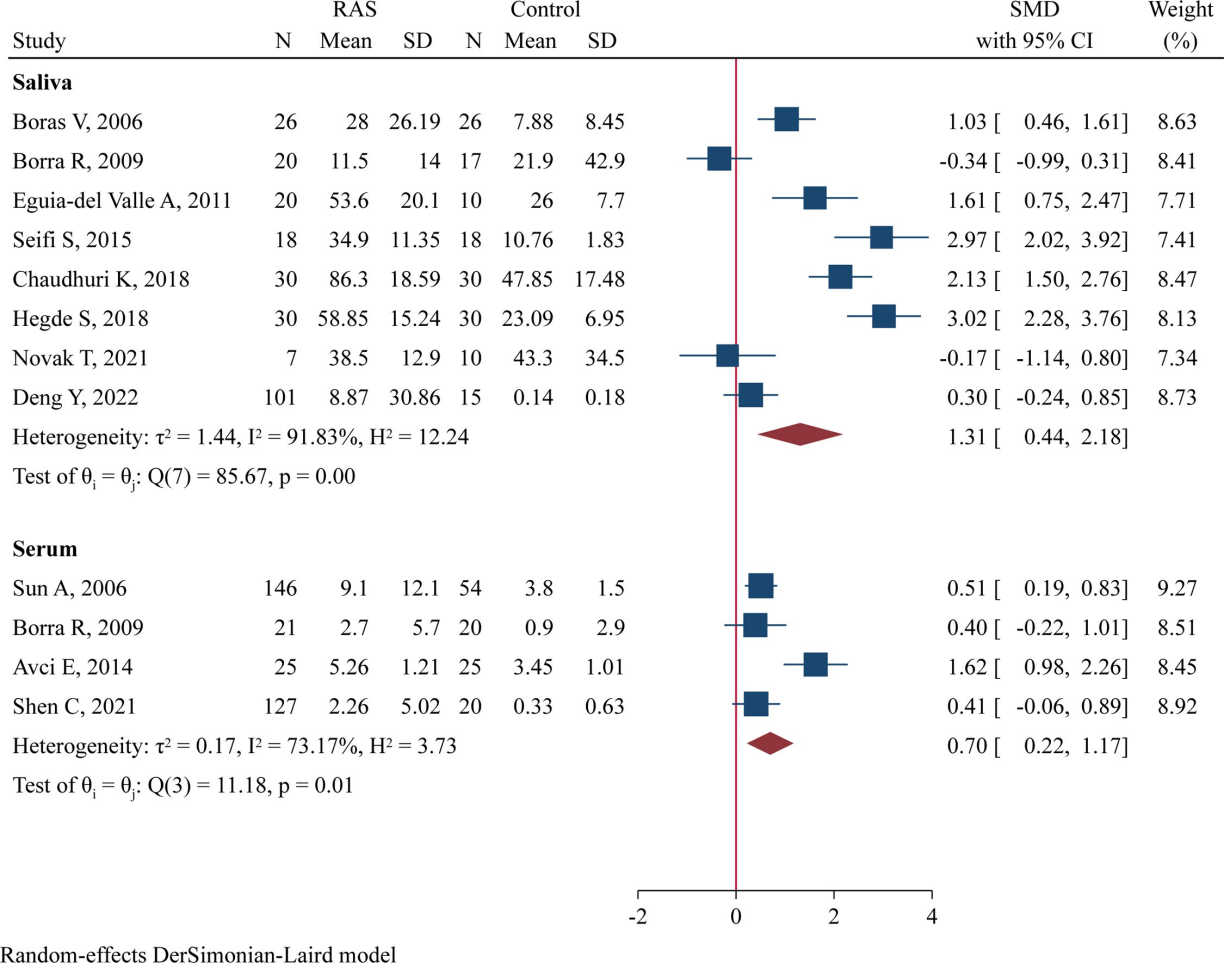
Forest plot of meta-analysis comparing TNF-α levels in saliva and serum samples between cases and controls.

The salivary levels of TNF-α were investigated in 8 studies (252 RAS and 156 controls) [13, 25–27, 33–36], and the serum levels were determined in 4 studies (319 RAS and 119 controls) [16, 26, 32, 37]. Compared to controls, RAS patients had significantly higher salivary TNF-α concentrations (SMD = 1.31, 95%CI 0.44–2.18, P = 0.003; Fig 5) and serum TNF-α levels (SMD = 0.70, 95%CI 0.22–1.17, P = 0.004; Fig 5). The effect size for concentration difference in saliva samples was large, while that in serum samples was moderate.

### IFN-γ levels

**Fig 6:**
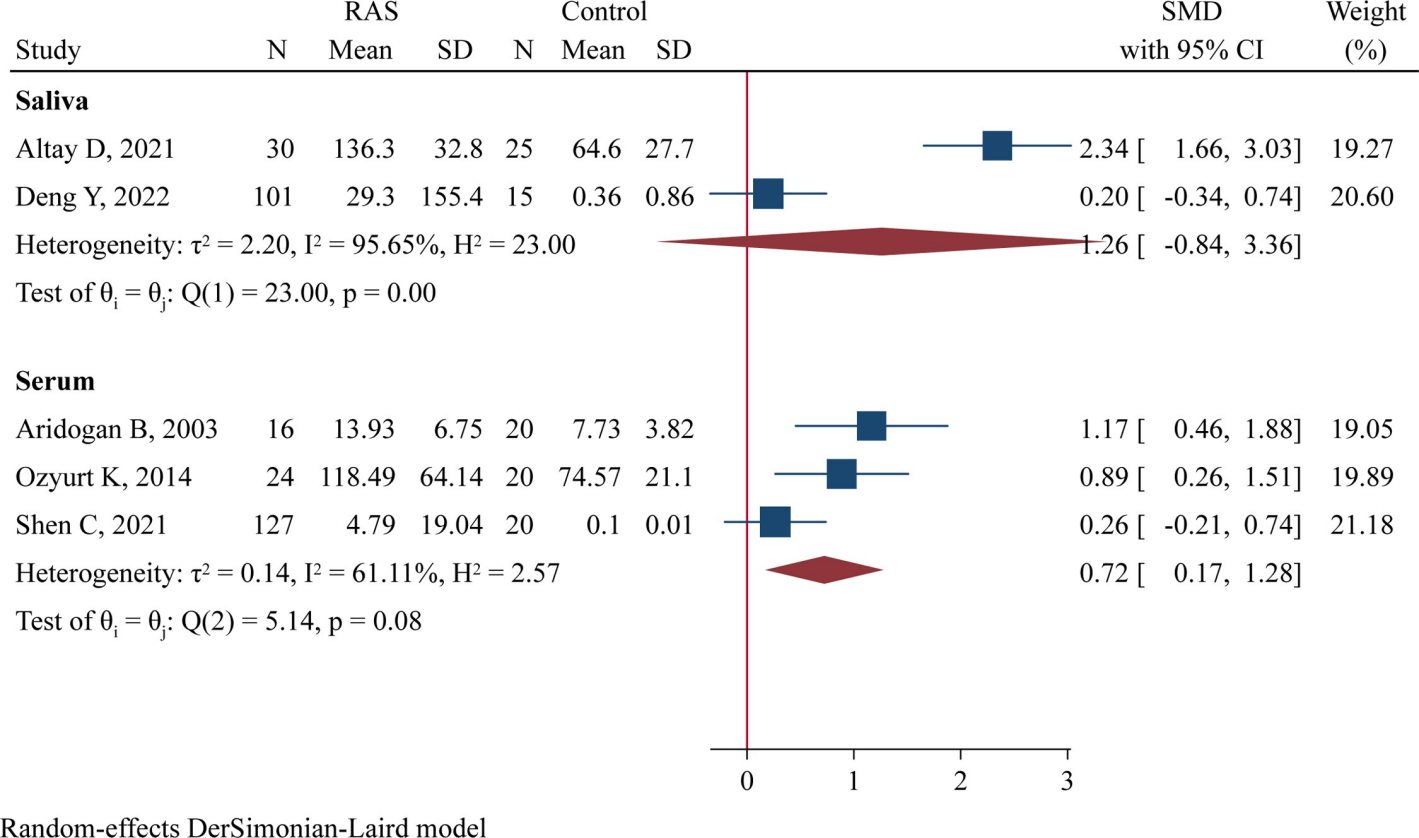
Forest plot of meta-analysis comparing IFN-γ levels in saliva and serum samples between cases and controls.

One study was excluded from quantitative synthesis because IFN-γ was below the detection level in most samples [36]. Thus, 5 studies with a total of 298 cases and 100 controls were included. Among them, two studies comprising 131 RAS and 40 cases explored the difference in salivary IFN-γ levels [12, 35], while three studies including 167 RAS and 60 controls evaluated the difference in serum IFN-γ concentrations [24, 29, 37]. Quantitative analysis using a random-effect model found significantly higher serum IFN-γ levels in RAS patients compared to healthy controls (SMD = 0.72, 95%CI 0.17–1.28, P = 0.010; Fig 6). However, salivary IFN-γ concentrations were comparable between both groups (SMD = 1.26, 95%CI -0.84 to 3.36, P = 0.240; Fig 6).

### Levels of other cytokines

Meta-analysis could not be conducted for IL-1 since only one study was available for each sample type [29, 36]. One study showed significantly higher serum IL-1 levels in RAS patients than healthy controls [29], whereas another study showed no significant difference in salivary IL-1 levels between the two groups [36]. Salivary IL-4 levels did not significantly differ between RAS patients and controls (S1 Fig). RAS patients had markedly increased salivary IL-5 concentrations than controls (S2 Fig). Serum IL-8 levels were comparable between the two groups (S3 Fig). Meta-analysis showed increased salivary IL-12 levels in RAS patients (S4 Fig). No significant differences were observed between RAS and controls in serum IL-13 levels (S5 Fig) or in salivary and serum levels of IL-17A (S6 Fig).

### Subgroups analysis

Subgroups analyses of these cytokines, stratified by RAS type, disease stage, and detection method, were further performed separately for saliva samples and serum samples. Meta-analysis were conducted only in subgroups with two or more available studies. In saliva samples (Table 2), IL-6 and TNF-α levels were significantly increased in patients with minor RAS and those in active phase compared to controls. In serum samples (Table 3), IL-6 levels were increased in patients with minor, major, and herpetiform RAS compared to controls.

**Table 2:** Subgroup analyses in saliva samples stratified by RAS type, disease stage and detection method.

Subgroups ^a^	No of studies	Sample size ^b^	I^2^ (%)	SMD	95%CI	P ^c^
Minor RAS						
IL-6	2	127/41	47.2	0.49	0.10, 0.88	0.013
TNF-α	3	145/59	91.3	1.38	0.08. 2.68	0.038
Active stage						
IL-6	2	127/41	47.2	0.49	0.10, 0.88	0.013
TNF-α	4	165/69	59.5	0.84	0.33, 1.35	0.001
ELISA method						
IL-2	4	87/85	97.2	4.15	0.83, 7.48	0.015
IL-12	2	27/30	42.2	0.94	0.18, 1.71	0.016
TNF-α	7	151/141	91.8	1.46	0.48, 2.44	0.003

^a^ Only subgroups with 2 or more available studies were analyzed.

^b^ Indicating the sample sizes of RAS and controls.

^c^ Comparison to healthy controls.

**Table 3:** Subgroup analyses in serum samples stratified by RAS type, disease stage, and detection method.

Subgroups ^a^	No of studies	Sample size ^b^	I^2^ (%)	SMD	95%CI	P ^c^
Minor RAS						
IL-6	3	309/151	0	0.40	0.19, 0.60	<0.001
TNF-α	4	242/119	72.4	0.71	0.23, 1.20	0.004
Major RAS						
IL-6	2	129/131	52.8	0.78	0.41, 1.15	<0.001
Herpetiform RAS						
IL-6	2	32/131	0	0.86	0.46, 1.26	<0.001
ELISA method						
IL-6	2	343/131	0	0.46	0.26, 0.67	<0.001
IL-10	2	41/45	89.9	-2.25	-3.99, -0.52	0.011
TNF-α	3	192/99	80.6	0.81	0.14, 1.49	0.019
INF-γ	2	40/40	0	1.01	0.54, 1.48	<0.001
CBA method						
IL-6	2	157/35	0	0.53	0.15, 0.91	0.006

^a^ Only subgroups with 2 or more available studies were analyzed.

^b^ Indicating the sample sizes of RAS and controls.

^c^ Comparison to healthy controls.

### Direct comparison of RAS types and disease stages

**Fig 7:**
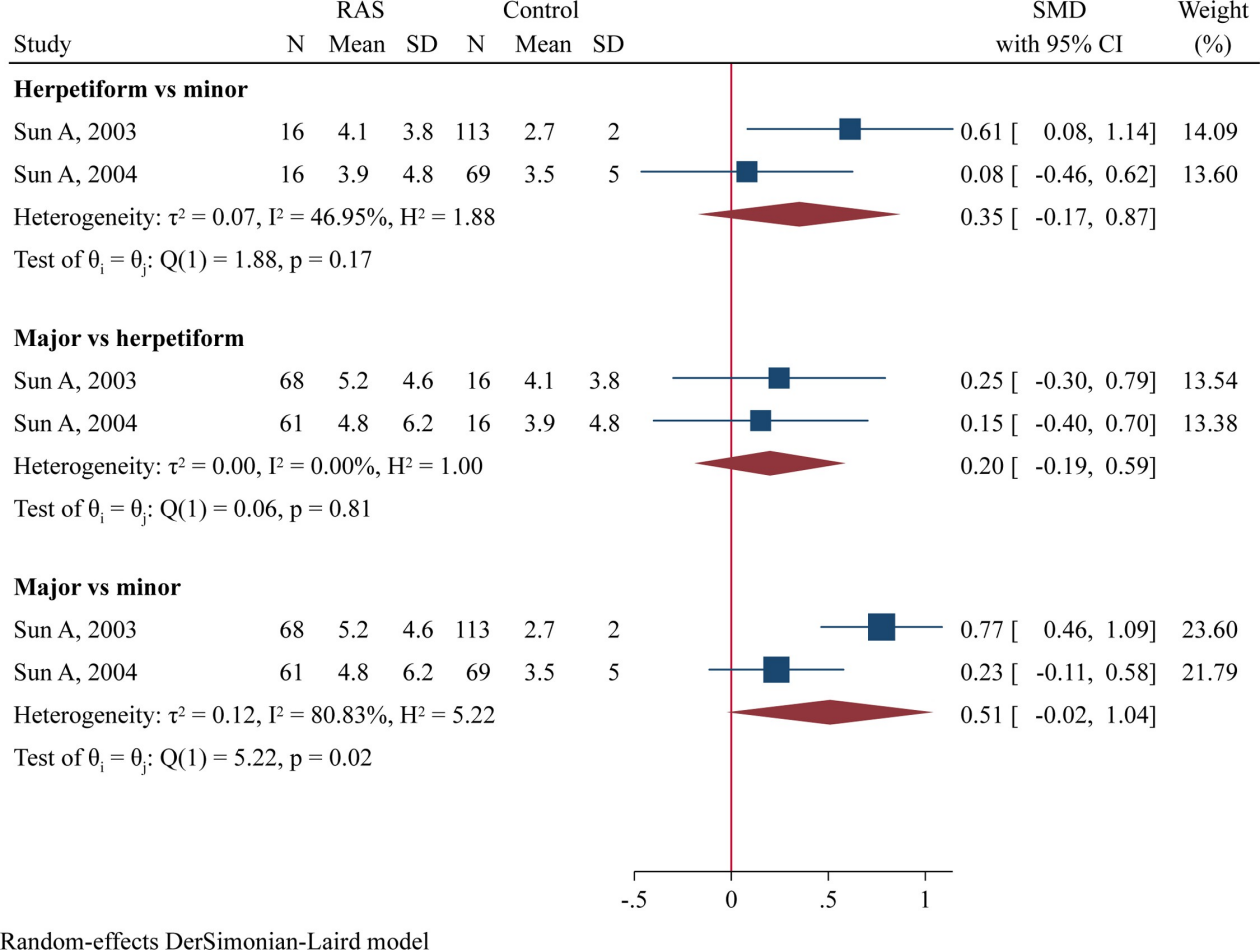
Forest plot of meta-analysis comparing serum IL-6 levels between different RAS types.

**Fig 8:**
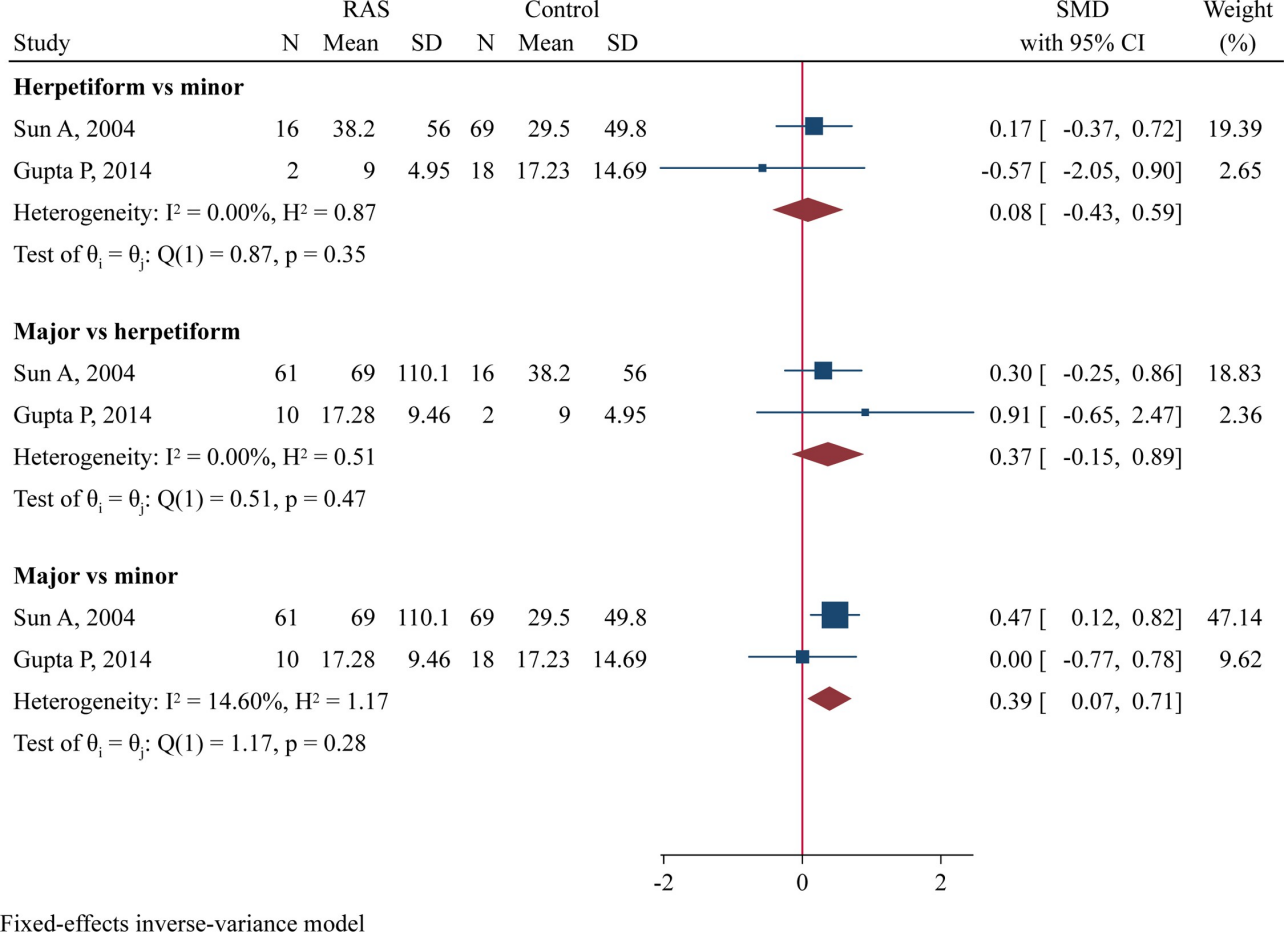
Forest plot of meta-analysis comparing serum IL-8 levels between different RAS types.

Two studies compared serum IL-6 levels between different RAS types [15, 31]. No significant differences were found when comparing herpetiform RAS to minor RAS or major RAS to herpetiform RAS (Fig 7). However, there was a trend toward higher serum IL-6 levels in major RAS compared to minor RAS (SMD = 0.51, 95%CI -0.02 to 1.04, P = 0.060; Fig 7). Additionally, two studies compared serum IL-8 levels between different RAS types [28, 31], finding significantly higher IL-8 levels in patients with major RAS than those with minor RAS (SMD = 0.39, 95%CI 0.07–0.71, P = 0.016; Fig 8). Two studies compared TNF-α levels in saliva samples collected during the active phase with those collected during the remission phase [25, 33], but no differences was observed between these two stages (S7 Fig).

### Sensitivity analysis and publication bias

Sensitivity analysis showed the omission of a single study did not have significant impact on pooled results. Potential publication bias was detected in the analysis of serum IL-2 and serum IFN-γ levels (S4 Table).

### Certainty of evidence

As shown in S5 Table, the results of meta-analyses for IL-2, IL-6, and TNF-α were deemed to have low or very low certainty of evidence. The major issues down-grading certainty included very serious inconsistency due to huge heterogeneity and serious indirectness due to the calculation of standard mean difference which did not represent the absolute differences in cytokine levels.

## Discussion

Th1/2/17-related cytokines are hypothesized to be involved in the pathogenesis of RAS, and their expression levels have been determined and compared between RAS patients and healthy controls. However, previous studies have yielded inconsistent results and the roles of these cytokines in RAS development and progression remain controversial. We performed the present meta-analysis by incorporating 20 studies that comprised 1070 RAS patients and 536 healthy controls. Our meta-analysis revealed significantly higher levels of various Th1 (IL-2, IL-12, TNF-α, IFN-γ) and Th2 (IL-5, IL-6)-related cytokines in RAS patients compared to controls. These results provide insights into the pathogenesis of RAS and suggest that T helper cell-related cytokines may be therapeutic targets.

IL-2 is secreted following the stimulation of various pathogens and drives Th1 immune response by promoting the activation and proliferation of Th1 cells [1, 38]. Increased IL-2 further stimulates the secretion of other pro-inflammatory cytokines, such as IL-1, IL-2, IL-12, TNF-α, and IFN-γ [1]. The secretion of IL-2 can be promoted by IL-6 through stimulating the proliferation of T helper and cytotoxic T cells [39]. IL-6 is mainly expressed by T cells, B cells, and macrophages. It stimulates B cell proliferation and T cell differentiation, acting as a key component of the immune and inflammatory response [40]. Excessive release of IL-6 activates multiple immune response processes, aggravates inflammatory mucosal lesions, and worsens oral ulcers [41]. TNF-α promotes the release of pro-inflammatory cytokines and inhibits anti-inflammatory cytokines. Furthermore, TNF-α stimulates the expression of major histocompatibility (MHC) I and II antigens on epithelial cells from the pre-ulcerative phase to ulcerative phase [42]. Subsequently, T lymphocytes recognize these cells, triggering a cytotoxic response and causing oral ulceration [42]. Besides Th1/Th2-related cytokines, IL-17A, a Th17-related cytokine, has also been associated with many autoimmune and inflammatory diseases [43]. Enhanced expression of IL-17A may cause excessive recruitment of lymphocytes and inflammatory cells, leading to tissue inflammation and damage [44]. IL-17A and TNF-α also exert an synergistic effect on keratinocytes and inflammatory cells [45]. Our meta-analysis demonstrates significantly increased levels of several Th1/Th2-related cytokines in RAS patients compared with healthy controls. Excessive release of these cytokines disrupts the microenvironment balance of T lymphocyte-mediated immune responses, causes aggregation of various immune cells and cytokines in local tissues, and contributes to the pathogenesis of RAS.

IL-10 is a key anti-inflammatory cytokine that inhibits the differentiation of T helper cells to Th1 cells, the migration of inflammatory cells, and the release of cytokines [46]. IL-10 also stimulates the proliferation and maturation of B cells, which promotes a Th2 response [46]. Therefore, altered IL-10 expression may contribute to an imbalance in the Th1/Th2 immune response. However, several studies have reported conflicting results when comparing IL-10 levels between RAS patients and controls. Aridogan B *et al*. and Avci E *et al*. detected reduced serum IL-10 levels, while Bhosale S *et al*. found reduced salivary IL-10 level in RAS patients [16, 17, 24]. Conversely, Novak T *et al*. and Deng Y *et al*. observed elevated salivary IL-10 levels in RAS patients [35, 36]. Fine regulation of IL-10 expression may be crucial for maintaining the balance of Th1 and Th2 immune responses and preventing the occurrence of oral ulcerations. However, the exact role of IL-10 in the development of RAS requires further investigation.

Cytokine levels may correlate with RAS severity and disease phases. Sun A *et al*. found that major and herpetiform types of RAS had significantly increased serum levels of IL-6 than minor RAS [15]. Another study by the same researchers revealed elevated TNF-α levels in major RAS compared to minor RAS [32]. Boras V *et al*. detected higher TNF-α levels in saliva samples collected during the remission phase than in samples collected during the acute phase among minor RAS patients, suggesting a more important role for TNF-α in the healing period than previously thought [25]. Conversely, Seifi S *et al*. found a slightly higher, but not significantly elevated, concentration of TNF-α in the acute phase compared to the remission phase [33]. The present meta-analysis, for the first time, compared cytokine levels between patients with difference RAS types and patients in different disease phases. We found that major RAS patients had higher serum IL-8 levels (P = 0.016) and a trend toward higher serum IL-6 levels (P = 0.060) than minor RAS patients, suggesting a role for IL-6 and IL-8 in RAS progression. However, no significant differences in IL-6 and IL-8 levels were found when comparing major RAS to herpetiform RAS. The involvement of cytokines in the development of more severe RAS and in the healing process of ulcers remains unclear and requires further investigation.

There were several limitations in our study. Firstly, the total sample size was small, as most cytokines were investigated by only a few studies. Secondly, there was huge between-study heterogeneity and significant variation in cytokine levels due to differences in disease severity, disease phage, sample collection, and detection method among included studies. Thirdly, the certainty of evidence of our results was low or very low according to the GARDE assessment.

## Conclusions

The present meta-analysis demonstrates significantly altered expression levels of Th1/Th2/Th17-related cytokines in RAS patients compared to healthy controls. Our results suggest that these cytokines, especially IL-2, IL-6, and TNF-α, are involved in the pathogenesis of RAS development and progression and are potential therapeutic targets for RAS.

## Supporting information

S1 Checklist PRISMA 2020 Checklist. (DOCX)

S1 Table Search strategy for each electronic database. (DOCX)

S2 Table Reasons of study exclusion. (DOCX)

S3 Table Quality assessment of included studies by Newcastle-Ottawa scale. (DOCX)

S4 Table Egger’s test for publication bias. (DOCX)

S5 Table Summary of the certainty of evidence using grading of recommendation assessment, development and evaluation approach. (DOCX)

S1 Fig Forest plot of meta-analysis comparing IL-4 levels between cases and controls. (PDF)

S2 Fig Forest plot of meta-analysis comparing IL-5 levels between cases and controls. (PDF)

S3 Fig Forest plot of meta-analysis comparing IL-8 levels between cases and controls. (PDF)

S4 Fig Forest plot of meta-analysis comparing IL-12 levels between cases and controls. (PDF)

S5 Fig Forest plot of meta-analysis comparing IL-13 levels between cases and controls. (PDF)

S6 Fig Forest plot of meta-analysis comparing IL-17A levels between cases and controls. (PDF)

S7 Fig Forest plot of meta-analysis comparing salivary TNF-α levels between active phase and remission phase. (PDF)
