# Supplementary material for: Evaluation of cytokine expressions in patients with recurrent aphthous stomatitis: A systematic review and meta-analysis
Source: PLoS One. 2024 Jun 11;19(6):e0305355. doi: 10.1371/journal.pone.0305355 (PMC11166324; doi:10.1371/journal.pone.0305355)
Supplement: S1 Table — (DOCX) [file pone.0305355.s002.docx]

Table S1. Search strategy for each electronic database.

| Databases | Search terms | No. of retrieved items |
| --- | --- | --- |
| PubMed | (‘Stomatitis, Aphthous’[MeSH] OR ‘recurrent oral ulcer*’[Title/Abstract] OR ‘recurrent aphthous ulcer*’ [Title/Abstract] OR ‘recurrent aphthous stomatitis’ [Title/Abstract]) AND (‘Interleukins’[MeSH] OR ‘interleukin*’ [Title/Abstract] OR ‘Tumor Necrosis Factor-alpha’[MeSH] OR ‘tumor necrosis factor*’[Title/Abstract] OR ‘Cytokines’[MeSH] OR ‘cytokine*’[Title/Abstract] OR ‘Interferons’[MeSH] OR ‘interferon*’[Title/Abstract]) | 294 |
| EMBASE | (‘recurrent oral ulcer*’ OR ‘recurrent aphthous ulcer*’ OR ‘recurrent aphthous stomatitis’) AND (‘interleukin*’ OR ‘tumor necrosis factor*’ OR ‘cytokine*’ OR ‘interferon*’) | 306 |
| Web of Science | (‘recurrent oral ulcer*’ OR ‘recurrent aphthous ulcer*’ OR ‘recurrent aphthous stomatitis’) AND (‘interleukin*’ OR ‘tumor necrosis factor*’ OR ‘cytokine*’ OR ‘interferon*’) | 388 |
| Google Scholar | (‘recurrent oral ulcer*’ OR ‘recurrent aphthous ulcer*’ OR ‘recurrent aphthous stomatitis’) AND (‘interleukin*’ OR ‘tumor necrosis factor*’ OR ‘cytokine*’ OR ‘interferon*’) | First 300 items sorted by relevance |
