# Supplementary material for: Evaluation of cytokine expressions in patients with recurrent aphthous stomatitis: A systematic review and meta-analysis
Source: PLoS One. 2024 Jun 11;19(6):e0305355. doi: 10.1371/journal.pone.0305355 (PMC11166324; doi:10.1371/journal.pone.0305355)
Supplement: S2 Table — (DOCX) [file pone.0305355.s003.docx]

S2 Table. Reasons of study exclusion.

| Study | Title | Exclusion reason |
| --- | --- | --- |
| Albanidou-Farmaki E, 2007 | Detection, enumeration and characterization of T helper cells secreting type 1 and type 2 cytokines in patients with recurrent aphthous stomatitis | Enumerate T cells secreting cytokines |
| Alpay-Kanitez N, 2015 | Salivary IL-1 alpha and IL-1 beta levels associated with oral mucosal disease activity in behcet's disease | Meeting abstract |
| Cui J, 2022 | Clinical Evaluation and Therapeutic Effects of Combination Treatment with Mecobalamin + Vitamin E in Recurrent Oral Ulcer | No healthy control |
| Curnow S, 2008 | Serum cytokine profiles in Behçet's disease: is there a role for IL-15 in pathogenesis? | Data not available |
| Dalghous A, 2006 | Expression of cytokines, chemokines, and chemokine receptors in oral ulcers of patients with Behcet's disease (BD) and recurrent aphthous stomatitis is Th1-associated, although Th2-association is also observed in patients with BD | Detect tissue expression |
| Deng Y, 2022 | A Randomized controlled clinical trial on dose optimization of thalidomide in maintenance treatment for recurrent aphthous stomatitis | No healthy control |
| Elamrousy W, 2021 | Evaluation of Novel Topical Camel Whey Protein Gel for the Treatment of Recurrent Aphthous Stomatitis: ,,,,Randomized Clinical Study | No healthy control |
| Han Y, 2021 | LncRNA NEAT1 is upregulated in recurrent aphthous stomatitis (RAS) and has predictive values | Data not available |
| Lewkowicz N, 2005 | Predominance of Type 1 cytokines and decreased number of CD4(+)CD25(+high) T regulatory cells in peripheral blood of patients with recurrent aphthous ulcerations | Detect cytokine expression level in PBMC culture |
| Lin S, 2005 | Study of the viral infections and cytokines associated with recurrent aphthous ulceration | Data not available |
| Lu J, 2020 | LncRNA CASC 2 is upregulated in aphthous stomatitis and predicts the recurrence | Data not available |
| Mimura M, 2017 | Immune response of patients with recurrent aphthous stomatitis challenged with a symbiotic | Data not available |
| Sun A, 2000 | Expression of interleukin-2 receptor by activated peripheral blood lymphocytes upregulated by the plasma level of interleukin-2 in patients with recurrent aphthous ulcers | No full text |
| Taylor L, 1992 | Increased production of tumour necrosis factor by peripheral blood leukocytes in patients with recurrent oral aphthous ulceration | Detect cytokine expression level in PBMC culture |
| Yamamoto Y, 1994 | Serum cytokines, interleukin-2 receptor, and soluble intercellular adhesion molecule-1 in oral disorders | Data not available |
| Zhu S, 2018 | Curative effect of oral ulcer powder on the treatment of recurrent aphthous ulcer | No healthy control |
