# Supplementary material for: Evaluation of cytokine expressions in patients with recurrent aphthous stomatitis: A systematic review and meta-analysis
Source: PLoS One. 2024 Jun 11;19(6):e0305355. doi: 10.1371/journal.pone.0305355 (PMC11166324; doi:10.1371/journal.pone.0305355)
Supplement: S3 Table — (DOCX) [file pone.0305355.s004.docx]

S3 Table. Quality assessment of included studies by Newcastle-Ottawa Scale.

| Study | Selection | | | | Comparability | | Exposure | | | Total |
| --- | --- | --- | --- | --- | --- | --- | --- | --- | --- | --- |
|  | 1 | 2 | 3 | 4 | 5a | 5b | 6 | 7 | 8 |  |
| Sun A, 2003 [15] | * | * |  | * |  | * | * | * | * | 7 |
| Aridogan B, 2003 [24] | * | * |  | * | * | * | * | * | * | 8 |
| Sun A, 2004 [31] | * | * |  | * |  | * | * | * | * | 7 |
| Sun A, 2006 [32] | * | * |  | * |  | * | * | * | * | 7 |
| Boras V, 2006 [25] | * | * | * | * | * |  | * | * | * | 8 |
| Borra R, 2009 [26] | * | * | * | * | * | * | * | * | * | 9 |
| Eguia-del Valle A, 2011 [27] | * | * | * | * |  |  | * | * | * | 7 |
| Pekiner F, 2012 [30] | * | * | * | * | * | * | * | * | * | 9 |
| Avci E, 2014 [16] | * | * |  | * | * | * | * | * | * | 8 |
| Ozyurt K, 2014 [29] | * | * | * | * | * | * | * | * | * | 9 |
| Gupta P, 2014 [28] | * | * | * | * | * | * | * | * | * | 9 |
| Kalpana R, 2014 [14] | * | * | * | * | * |  | * | * | * | 8 |
| Seifi S, 2015 [33] | * | * |  | * | * | * | * | * | * | 8 |
| Chaudhuri K, 2018 [34] | * | * |  | * | * | * | * | * | * | 8 |
| Bhosale S, 2018 [17] | * | * |  | * | * | * | * | * | * | 8 |
| Hegde S, 2018 [13] | * | * |  | * | * | * | * | * | * | 8 |
| Shen C, 2021 [37] | * | * | * | * | * | * | * | * | * | 9 |
| Altay D, 2021 [12] | * | * |  | * | * | * | * | * | * | 8 |
| Novak T, 2021 [36] | * | * |  | * | * | * | * | * | * | 8 |
| Deng Y, 2022 [35] | * | * | * | * | * | * | * | * | * | 9 |

Selection domain: (1) Is the case definition adequate?; (2) Representativeness of the cases; (3) Selection of Controls; (4): Definition of Controls.

Comparability domain: (5a) Matched for age; (5b) Matched for gender.

Exposure domain: (6) Ascertainment of exposure; (7) Same method of ascertainment for cases and controls; (8) Non-Response rate
