# Supplementary material for: Evaluation of cytokine expressions in patients with recurrent aphthous stomatitis: A systematic review and meta-analysis
Source: PLoS One. 2024 Jun 11;19(6):e0305355. doi: 10.1371/journal.pone.0305355 (PMC11166324; doi:10.1371/journal.pone.0305355)
Supplement: S4 Table — (DOCX) [file pone.0305355.s005.docx]

S4 Table. Egger’s test for publication bias

| Cytokines | No. of studies | z | P |
| --- | --- | --- | --- |
| IL-2 (saliva) | 4 | 1.52 | 0.129 |
| IL-2 (serum) | 3 | 2.14 | 0.032 |
| IL-6 (saliva) | 3 | -0.13 | 0.898 |
| IL-6 (serum) | 4 | 0.29 | 0.774 |
| IL-10 (saliva) | 3 | -0.05 | 0.962 |
| TNF-α (saliva) | 8 | 0.64 | 0.522 |
| TNF-α (serum) | 4 | 0.89 | 0.373 |
| INF-γ (serum) | 3 | 2.26 | 0.024 |
