# Supplementary material for: Evaluation of cytokine expressions in patients with recurrent aphthous stomatitis: A systematic review and meta-analysis
Source: PLoS One. 2024 Jun 11;19(6):e0305355. doi: 10.1371/journal.pone.0305355 (PMC11166324; doi:10.1371/journal.pone.0305355)
Supplement: S5 Table — (DOCX) [file pone.0305355.s006.docx]

S5 Table Summary of the certainty of evidence using Grading of Recommendation Assessment, Development and Evaluation approach.

| No. of studies | Certainty assessment | | | | | |  | Effect | | Certainty |
| --- | --- | --- | --- | --- | --- | --- | --- | --- | --- | --- |
|  | Study design | Risk of bias | Inconsistency | Indirectness | Imprecision | Publication bias |  | No. of individuals | SMD (95%CI) |  |
| Salivary IL-2 | | | | | |  |  |  |  |  |
| 4 | Case-control | Not serious | Very serious ^a^ | Serious ^b^ | Serious ^c^ | None |  | 172 | 4.15 (0.83, 7.48) | ⊕◯◯◯  Very low |
| Serum IL-2 | | | | | |  |  |  |  |  |
| 3 | Case-control | Not serious | Very serious ^a^ | Serious ^b^ | Not serious | Potential publication bias |  | 242 | 1.29 (-0.45, 3.03) | ⊕◯◯◯  Very low |
| Salivary IL-6 | | | | | |  |  |  |  |  |
| 3 | Case-control | Not serious | Not serious | Serious ^b^ | Serious ^c^ | None |  | 185 | 0.48 (0.12, 0.84) | ⊕◯◯◯  Very low |
| Serum IL-6 | | | | |  |  |  |  |  |  |
| 4 | Case-control | Not serious | Not serious | Serious ^b^ | Not serious | None |  | 666 | 0.48 (0.30, 0.66) | ⊕⊕◯◯  Low |
| Salivary TNF-α | | | | | |  |  |  |  |  |
| 8 | Case-control | Not serious | Very serious ^a^ | Serious ^b^ | Not serious | None |  | 408 | 1.31 (0.44, 2.18) | ⊕◯◯◯  Very low |
| Serum TNF-α | | | | | | |  |  |  |  |
| 4 | Case-control | Not serious | Very serious ^a^ | Serious ^b^ | Not serious | None |  | 438 | 0.70 (0.22, 1.17) | ⊕◯◯◯  Very low |

^a^ Substantial heterogeneity was observed.

^b^ Calculating standard mean difference rather than mean difference of cytokine levels.

^c^ The total number of subjects was less than 200.
