# Supplementary figures and images for: Evaluation of cytokine expressions in patients with recurrent aphthous stomatitis: A systematic review and meta-analysis
Source: PLoS One. 2024 Jun 11;19(6):e0305355. doi: 10.1371/journal.pone.0305355 (PMC11166324; doi:10.1371/journal.pone.0305355)

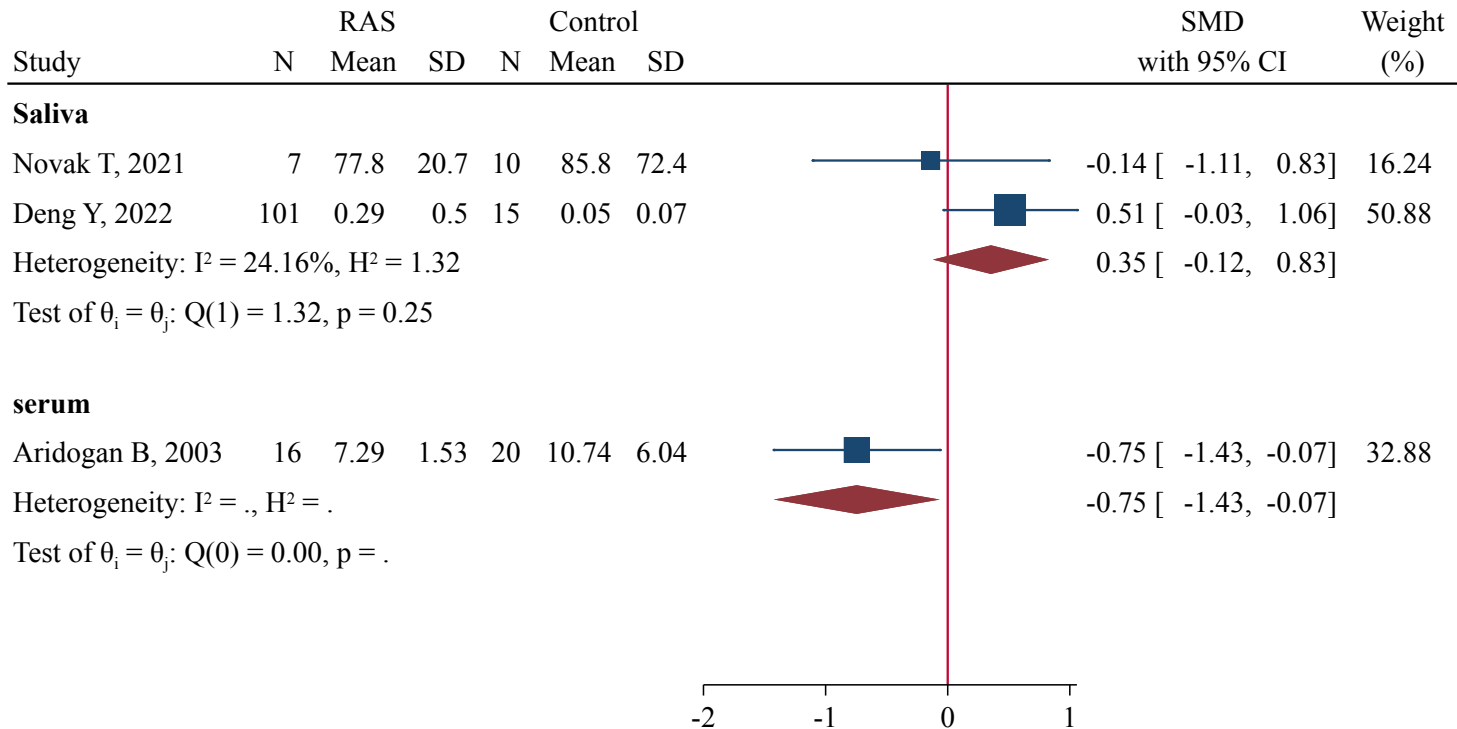

Fixed-effects inverse-variance model

Supplement: S1 Fig — (PDF) [file pone.0305355.s007.pdf]

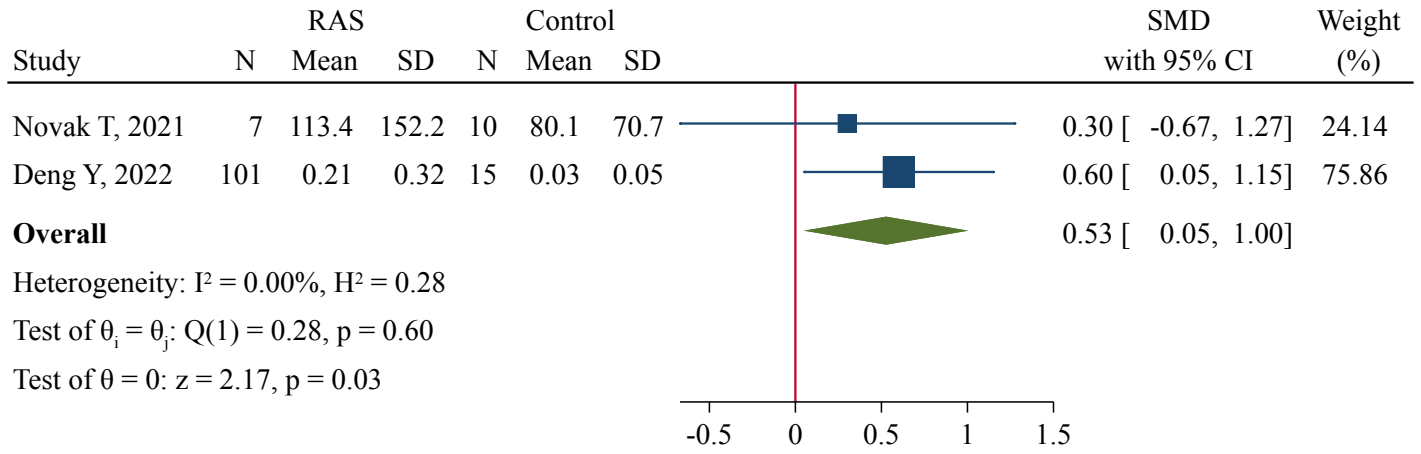

Fixed-effects inverse-variance model

Supplement: S2 Fig — (PDF) [file pone.0305355.s008.pdf]

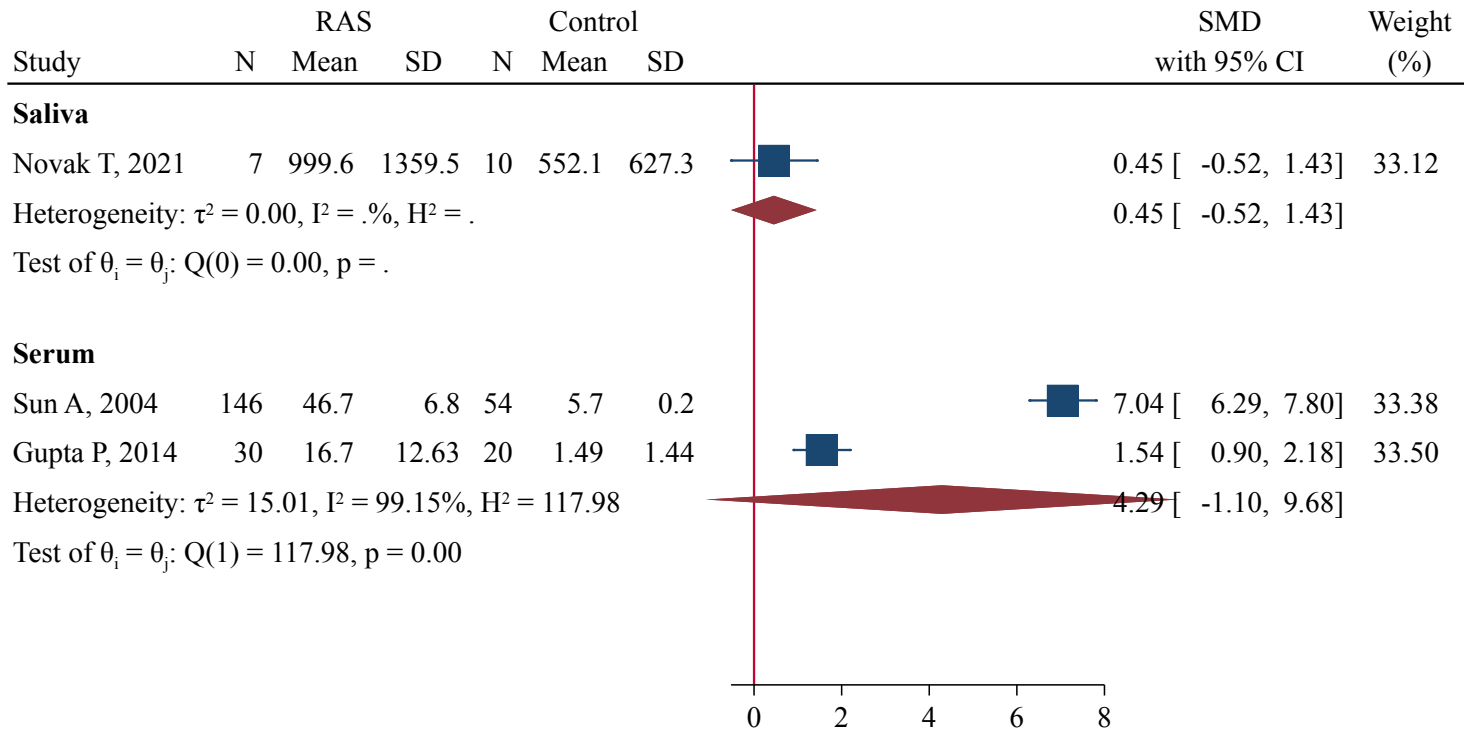

Supplement: S3 Fig — (PDF) [file pone.0305355.s009.pdf]

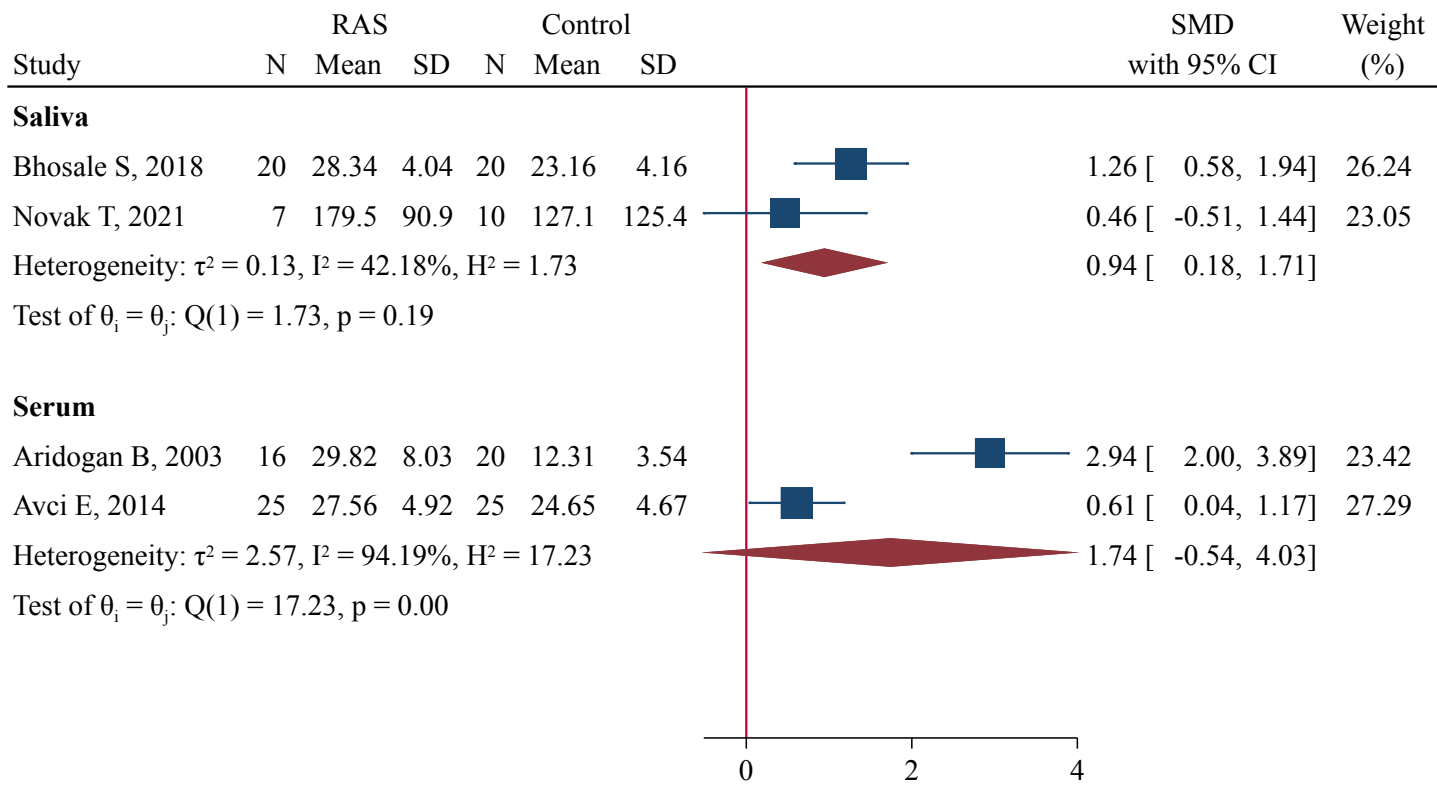

Random-effects DerSimonian-Laird model

Supplement: S4 Fig — (PDF) [file pone.0305355.s010.pdf]

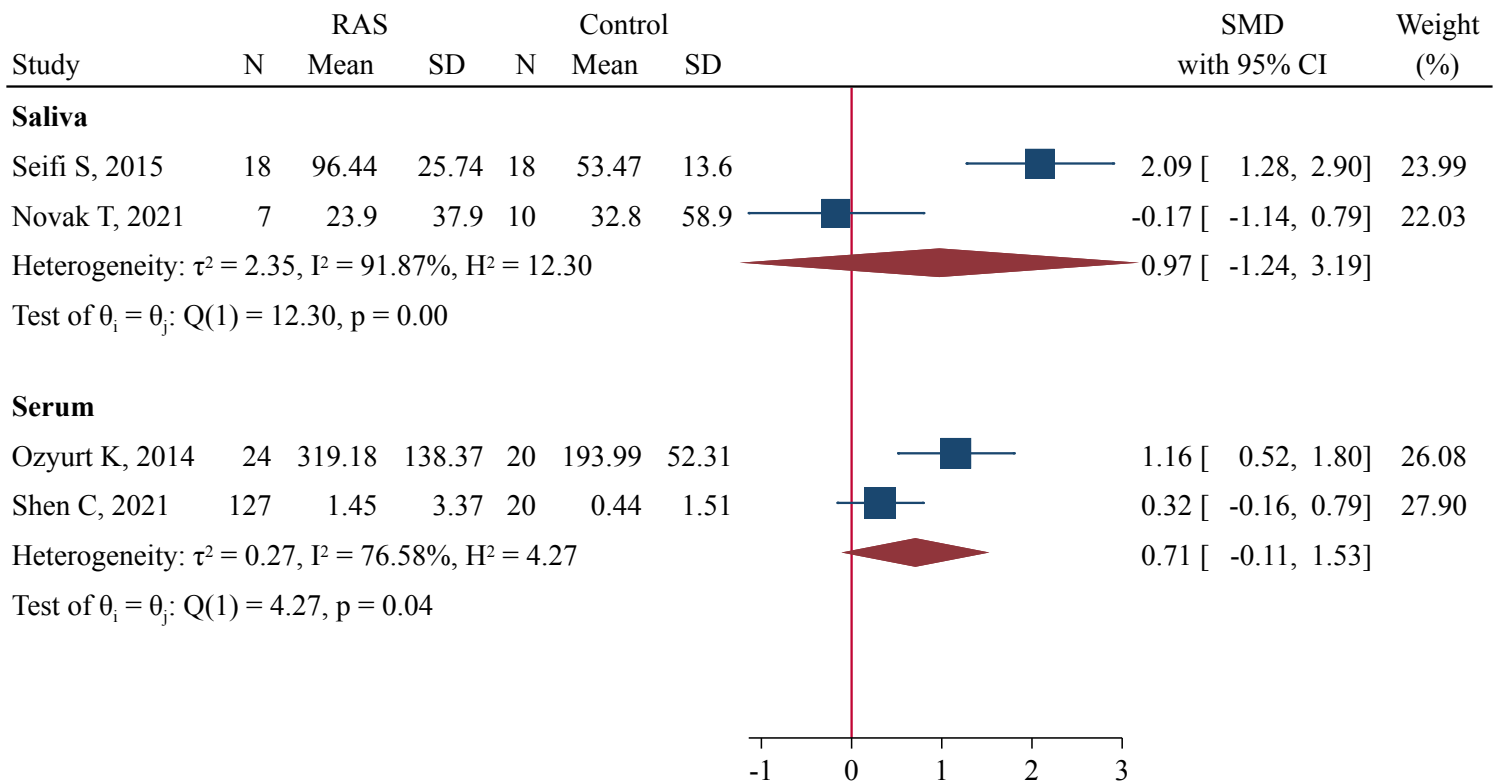

Random-effects DerSimonian-Laird model

Supplement: S6 Fig — (PDF) [file pone.0305355.s012.pdf]
