# Supplementary material for: Evaluation of cytokine expressions in patients with recurrent aphthous stomatitis: A systematic review and meta-analysis
Source: PLoS One. 2024 Jun 11;19(6):e0305355. doi: 10.1371/journal.pone.0305355 (PMC11166324; doi:10.1371/journal.pone.0305355)
Supplement: S5 Fig — (PDF) [file pone.0305355.s011.pdf]

Study	RAS N	RAS Mean	RAS SD	Control N	Control Mean	Control SD	SMD with 95% CI	Weight (%)
Aridogan B, 2003	16	11.82	3.85	20	28.27	15.88	-1.35 [-2.08, -0.63]	49.39
Ozyurt K, 2014	24	34.15	25.66	20	21.54	21.07	0.53 [-0.07, 1.14]	50.61
**Overall**							-0.40 [-2.25, 1.45]	

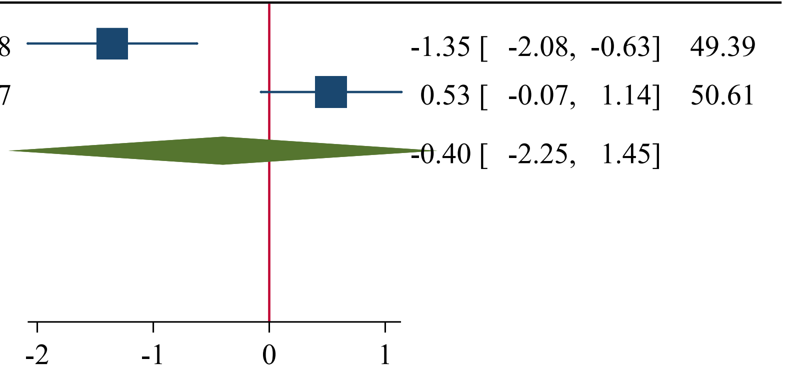

Heterogeneity: $\tau^2 = 1.66$, $I^2 = 93.46\%$, $H^2 = 15.29$

Test of $\theta_i = \theta_j$: $Q(1) = 15.29$, $p = 0.00$

Test of $\theta = 0$: $z = -0.42$, $p = 0.67$

Random-effects DerSimonian-Laird model
