# Supplementary material for: Evaluation of cytokine expressions in patients with recurrent aphthous stomatitis: A systematic review and meta-analysis
Source: PLoS One. 2024 Jun 11;19(6):e0305355. doi: 10.1371/journal.pone.0305355 (PMC11166324; doi:10.1371/journal.pone.0305355)
Supplement: S7 Fig — (PDF) [file pone.0305355.s013.pdf]

Study	RAS N	RAS Mean	RAS SD	Control N	Control Mean	Control SD	SMD with 95% CI	Weight (%)
Boras V, 2006	26	28	26.19	13	54.31	49.63	-0.74 [-1.43, -0.05]	49.87
Seifi S, 2015	18	34.9	11.35	18	28.09	9.07	0.66 [-0.01, 1.33]	50.13
**Overall**							-0.04 [-1.41, 1.34]	

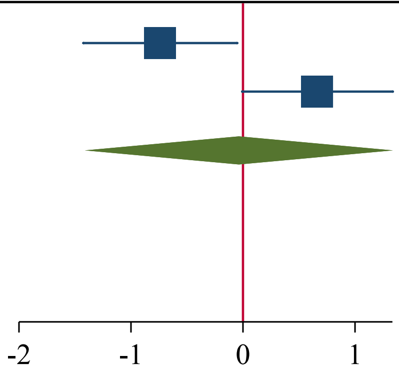

Heterogeneity: $\tau^2 = 0.86$, $I^2 = 87.83\%$, $H^2 = 8.22$

Test of $\theta_i = \theta_j$: Q(1) = 8.22, p = 0.00

Test of $\theta = 0$: z = -0.05, p = 0.96

Random-effects DerSimonian-Laird model
